# Radioprotective effects of Keratinocyte Growth Factor-1 against irradiation-induced salivary gland hypofunction

**DOI:** 10.18632/oncotarget.14583

**Published:** 2017-01-10

**Authors:** Jeong-Seok Choi, Hyun-Soo Shin, Hye-Young An, Young-Mo Kim, Jae-Yol Lim

**Affiliations:** ^1^ Department of Otorhinolaryngology-Head and Neck Surgery, Incheon, Republic of Korea; ^2^ Translational Research Center, Incheon, Republic of Korea

**Keywords:** salivary glands, irradiation, xerostomia, KGF-1, apoptosis

## Abstract

Irradiation can cause salivary gland hypofunction, with hyposalivation producing discomfort, health risks, and reducing function in daily life. Despite increasing translational research interest in radioprotection, there are no satisfactory treatments available. Keratinocyte growth factor-1 stimulates proliferation of salivary epithelial cells or salivary stem/progenitor cells. However, the exact mechanism of its radioprotection against radiation-induced salivary hypofunction is not fully elucidated. Our results reveal that the radioprotective effects of keratinocyte growth factor-1 involved alleviation of growth inhibition and anti-apoptotic cell death of human parotid epithelial cells. Furthermore, keratinocyte growth factor-1 protected human parotid epithelial cells through the phosphoinositide 3-kinase – protein kinase B (Akt) pathway and inhibition of p53-mediated apoptosis through activation of mouse double minute 2. Local delivery of keratinocyte growth factor-1 into the irradiated salivary glands could protect radiation-induced salivary cell damages, suppress p53-mediated apoptosis and prevent salivary hypofunction *in vivo*. This suggests that keratinocyte growth factor-1 is a promising candidate to prevent radiation-induced salivary hypofunction and raise rational development keratinocyte growth factor-1 local delivery system.

## INTRODUCTION

Radiotherapy is the main treatment in patients with head and neck cancer. Due to the structural proximity and complexity in head and neck area, irradiation-induced salivary gland (SG) damage is common. Hyposalivation-related complications such as xerostomia, halitosis, burning sensation, bizarre taste, swallowing difficulty and dental caries decrease the quality of life [1]. In spite of advances in SG-sparing radiation techniques, it remains challenging to prevent irradiation-induced salivary hypofunction.

Our previous study found that mesenchymal stem cell-secreted bioactive factors provide radioprotection and tissue remodeling [2]. An increasing number of studies using bioactive factors for prevention or amelioration of irradiation-induced SG damage have been conducted [3–6]. Keratinocyte growth factor-1 (KGF-1, also known as FGF-7) stimulates the growth of epithelial cells and protects those cells from chemotherapy or radiotherapy-induced oral mucositis. Recombinant KGF (Palifermin) has been approved as a cytoprotective drug to reduce the symptoms of oral mucositis [7, 8]. KGF-1 also has potential for amelioration of irradiation-induced salivary hypofunction [5, 6].

KGF-1 is a member of FGF7/10/22 subfamilies and acts exclusively through a subset of FGF receptors, FGFR2IIIb [9]. KGF-1 is related to epithelial cell growth, differentiation, survival, and DNA repair [10]. Although KGF-1 effectively protects and regenerates damaged epithelial cells in the gastrointestinal mucosa, lung and oral mucosa [9–14], the exact mechanisms are not understood, particularly in terms of radioprotection on epithelial cells of SGs.

In this study, we investigated the KGF-1 mechanism related to anti-apoptotic effect *in vitro* and determined whether the KGF-1 could prevent salivary hypofunction *in vivo*. Our results indicate for the first time that KGF-1 can provide radioprotection to SG epithelial cells by reducing DNA damage and p53-mediated apoptosis following irradiation, in which phosphoinositide 3-kinase (PI3K)- protein kinase B (Akt)-p53 pathway mediates the radioprotective effect of KGF-1.

## RESULTS

### Irradiation response of human parotid epithelial cells *in vitro*

**Figure 1:**
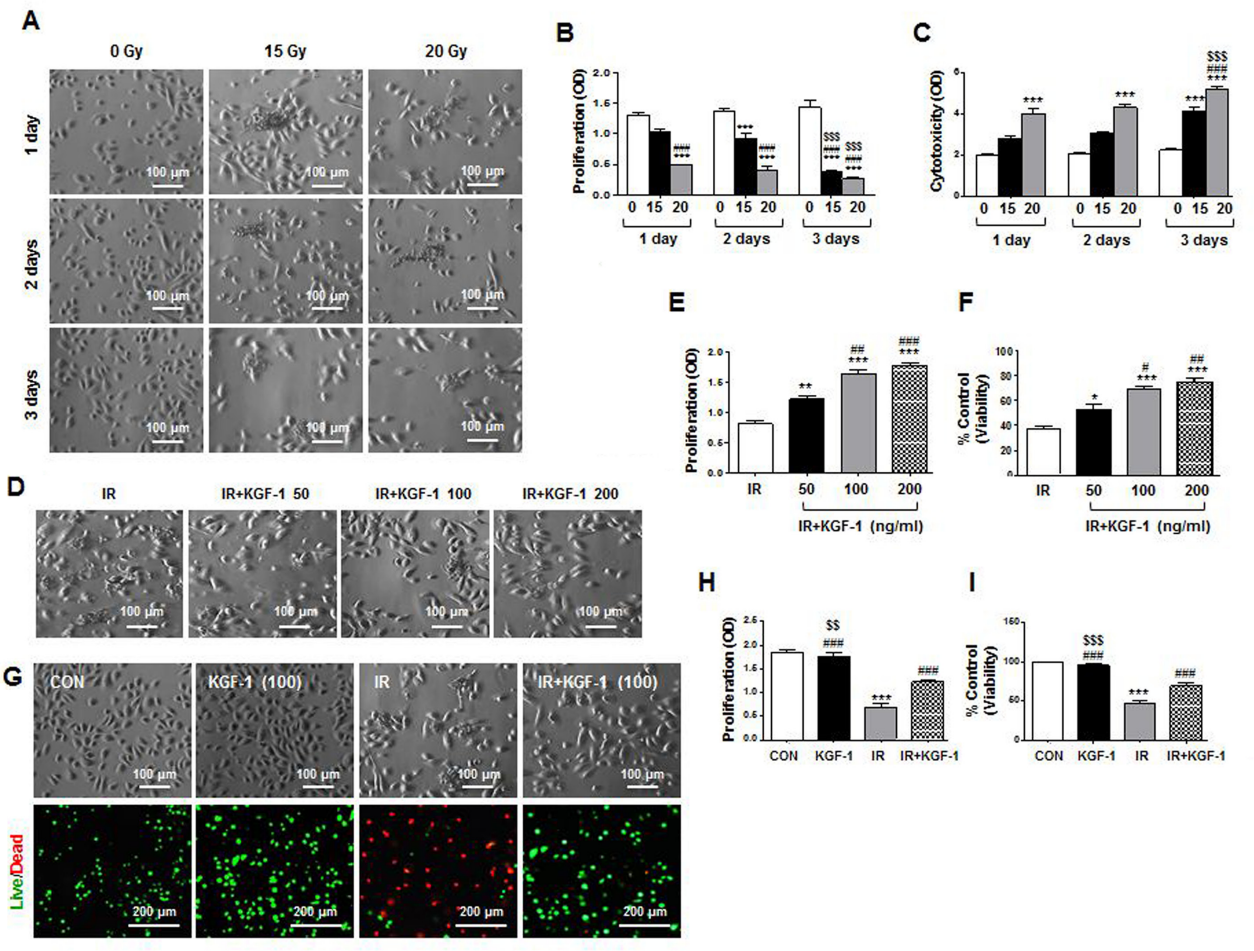
Morphological changes, cell proliferation and viability of hPECs after irradiation (**A**) Irradiation induced morphological changes of hPEC in a time- and dose-dependent manner. Scale bars represent 100 μm. (**B**) Proliferation of hPEC after irradiation was examined. (**C**) Cytotoxicity of hPEC after irradiation was examined. Data are presented as the means ± SEM (*n* = 5). Two-way ANOVA, Bonferroni's post hoc test. *, compared to 0Gy in each group; ^#^, compared to 15 Gy in each group, ^$^, compared to 15 and 20 Gy in 2 days. ***P <0.001, ^###^*P* < 0.001, ^$$$^*P* < 0.001. (**D**) Effect of dose dependent-KGF-1 on irradiation-induced changes in cell proliferation and viability in hPEC. Scale bars represent 100 μm. (**E**) Proliferation of hPEC after IR+KGF-1 was examined. (**F**) Cytotoxicity of hPEC after IR+KGF-1 was examined (*n* = 5). One-way ANOVA, Tukey's post hoc test. *, compared to IR; ^#^, compared to IR+KGF-1 (50 ng/ml). *P < 0.05, **P < 0.01, ***P < 0.001, ^#^*P* < 0.05, ^##^*P* < 0.01, ^###^*P* < 0.001. (**G**) Effect of KGF-1 on irradiation-induced changes in cell proliferation and viability in hPEC. Scale bars represent 100 and 200 μm. (**H**) Proliferation of hPEC after IR+KGF-1 was examined. (**I**) Cytotoxicity of hPEC after IR+KGF-1 was examined (*n* = 5). One-way ANOVA, Tukey's pot hoc test. *, compared to CON; ^#^, compared to IR; ^$^, compared to IR+KGF-1. ***P < 0.001, ^###^*P* < 0.001, ^$$^*P* < 0.01, ^$$$^*P* < 0.001.

We initially investigated irradiation response of human parotid epithelial cells (hPECs) *in vitro* to examine the mechanisms of KGF-1 on radioprotection of salivary epithelial cells. KGF-1 was administered immediately after irradiation. We assessed morphological changes, proliferation, and cytotoxicity of the monolayer cultured hPECs at one, two, and three days after irradiation at a dosage of 0, 15, and 20 Gy. Irradiation at a dosage of 15 and 20 Gy induced morphological changes of hPECs from a cuboidal, cobblestone appearance to destroyed, fibroblastoid morphology (Figure 1A). Irradiation significantly decreased proliferation and increased cytotoxicity by LDH release in the hPECs in a time dependent manner (Figure 1B and 1C). HPECs with 20 Gy of irradiation lost significant proliferative capacity while increasing LDH release from one day post-irradiation, suggesting an irradiation dose-response relationship.

KGF-1 at concentrations of 50, 100, and 200 ng/ml alleviated irradiation-induced growth inhibition and cytotoxic damage by irradiation at two days after irradiation (Figure 1D–1F). There was a more significant effect of 100 or 200 ng/ml of KGF-1 on irradiation-induced changes in cell proliferation and viability in hPECs than 50 ng/ml of KGF-1 (Figure 1E and 1F). In addition, 100 ng/ml of KGF-1 successfully reduced irradiation-induced growth inhibition and cell death by live/dead staining (Figure 1G–1I). KGF-1 itself did not affect cell proliferation or cell death. Based on these observations, 100 ng/ml of KGF-1 was chosen for further experiments.

**Figure 2:**
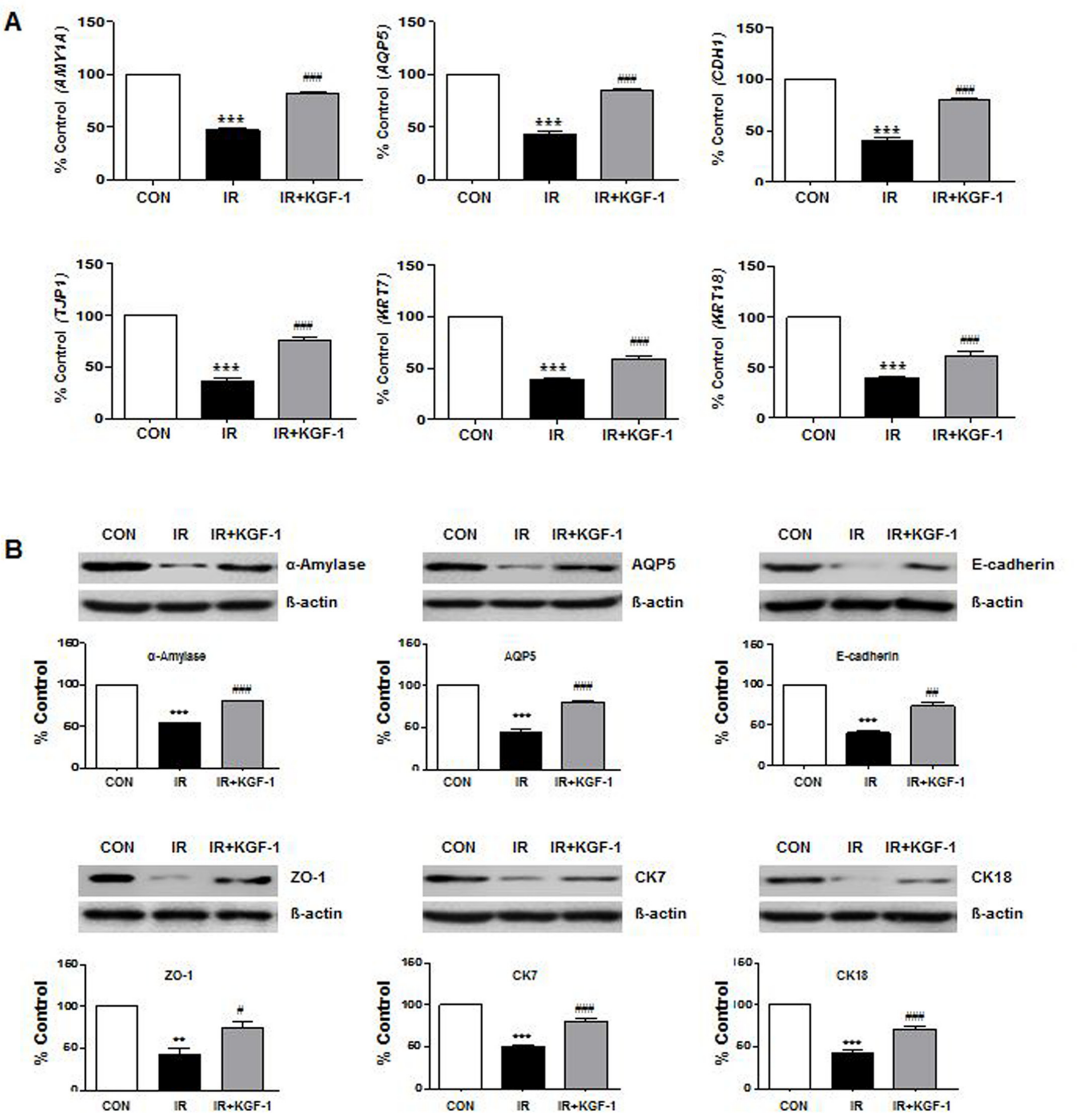
Effect of KGF-1 on salivary mRNA and protein expression (**A**) Irradiation significantly decreases the salivary acinar markers (α-amylase; *AMY1A* and AQP5; *AQP5*), TJ protein (ZO-1; *TJP1*), adherence protein (E-cadherin; *CDH1*), and ductal markers (CK7; *KRT7* and CK18; *KRT18*) in hPEC that is increased by treatment with KGF-1 (100 ng/ml) (*n* = 9). One-way ANOVA, Tukey's post hoc test. *, compared to CON; ^#^, compared to IR. ***P < 0.001, ^###^*P* < 0.001. (**B)** The protein translation of the same markers in Figure 2A was examined by Western blot, and the expression levels relative to β-actin were calculated (*n* = 3). One-way ANOVA, Tukey's post hoc test. *, compared to CON; ^#^, compared to IR. **P < 0.01, ***P < 0.001, ^#^*P* < 0.05, ^##^*P* < 0.01, ^##^#*P* < 0.001.

To investigate the phenotypic markers expression, mRNA and protein expression of acinar markers; α-amylase (*AMY1A*) and AQP5 (*AQP5*), ductal markers; CK7 (*KRT7*) and CK18 (*KRT18*), intercellular adherence protein; E-cadherin (*CDH1*), and TJ protein; ZO-1 (*TJP1*) were compared among the groups at two days after irradiation (Figure 2A and 2B). Irradiation reduced the salivary epithelial markers (α-amylase, AQP5, CK7, and CK18), ZO-1, and E-cadherin expression. KGF-1 treatment enhanced all the salivary epithelial markers and increased the ZO-1 and E-cadherin expression.

### Radioprotective mechanisms of KGF-1

**Figure 3:**
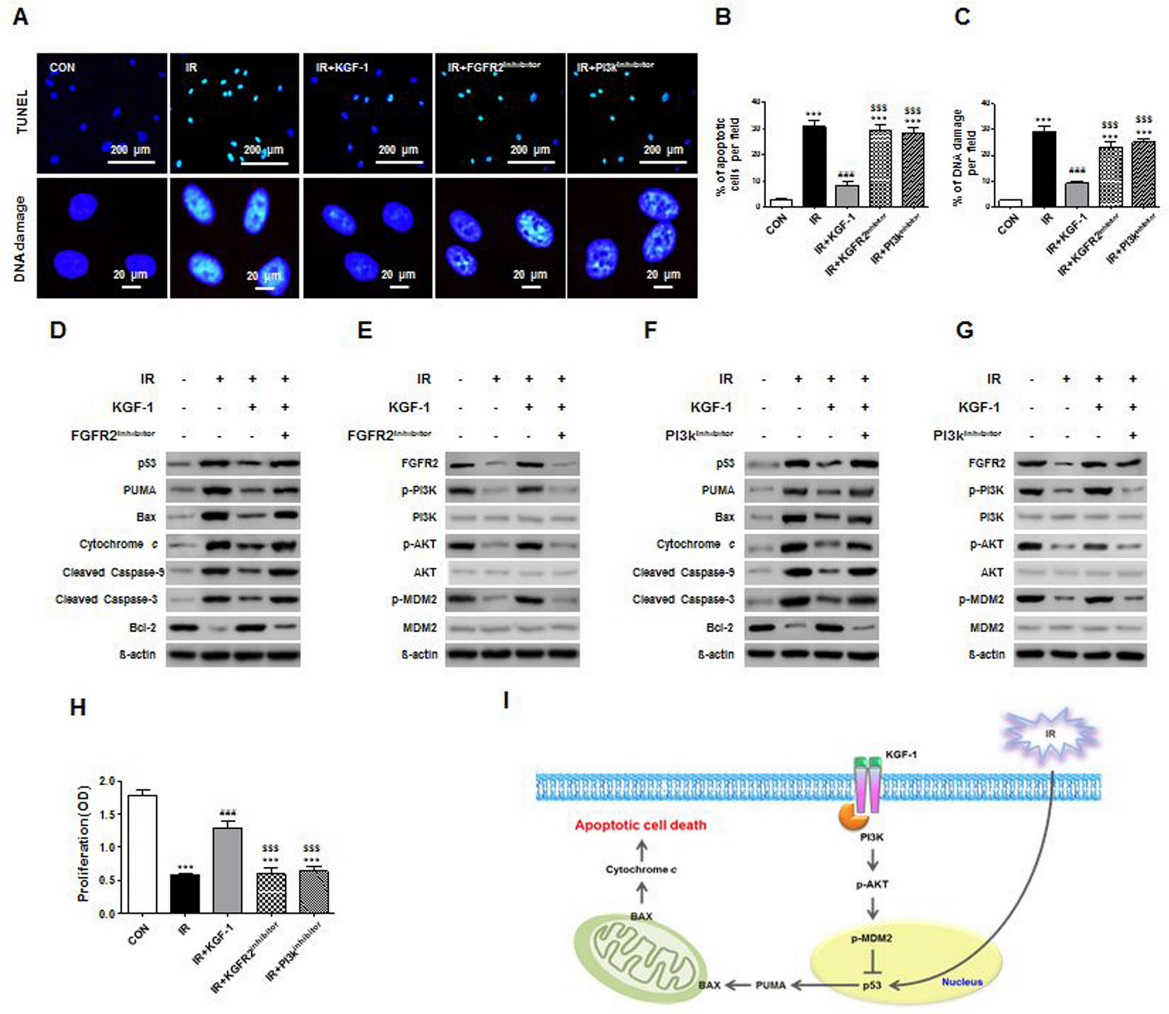
Effect of KGF-1 on apoptosis and apoptosis-related protein expression (**A**) KGF-1 effect of TUNEL positive cells and DNA damages in hPECs. (**B**) Comparison of the percentages of TUNEL-positive apoptotic cells among groups. (**C**) Comparison of the percentages of DNA damages among groups. Data are presented as the mean number of apoptotic cells per field ± SEM (*n* = 3). One-way ANOVA, Tukey's post hoc test. *, compared to CON; ^#^, compared to IR; ^$^, compared to IR+KGF-1. ***P < 0.001, ^###^*P* < 0.001, ^###^*P* < 0.001. (**D**–**G**) Effect of FGFR2 and PI3k inhibitor on irradiation-induced FGFR2-PI3k pathway and apoptosis. FGFR2 and PI3k inhibitor induces an increase of FGFR2-PI3k-related protein and pro-apoptotic protein in hPECs. (**H**) Comparison of the proliferation capacities among groups. (*n* = 5). One-way ANOVA, Tukey's post hoc test. *, compared to CON; ^#^, compared to IR, ^$^, compared to IR+ KGF-1. ***P < 0.001, ^###^*P* < 0.001, ^$$$^*P* < 0.001. (**I**) Proved signaling pathway of KGF-1 to block the apoptosis and DNA damage of hPECs after irradiation.

To understand the mechanism of irradiation-induced cell death, we performed an *in vitro* TUNEL assay, which revealed the presence of fragmented hPEC DNA. These findings are direct evidence of apoptotic cell death. Irradiation significantly increased DNA fragments and TUNEL-positive apoptotic cells and KGF-1 successfully reduced DNA fragments and TUNEL-positive apoptotic cells (Figure 3A). We investigated whether cell death was related to irradiation-induced DNA damage, and our results showed that DNA damage marker, γH2AX significantly decreased after KGF-1 treatment (Figure 3A). In addition, the radioprotective effect of KGF-1 against DNA damage and cell death was inhibited in the presence of FGFR2 inhibitor or PI3k inhibitor in the medium (Figure 3B–3C).

To analyze whether the radioprotective effect of KGF-1 is attributed to an anti-apoptotic effect, we examined the changes in apoptosis-associated proteins including p53, PUMA, Bax, cytochrome c, cleaved caspase-9 and -3, and Bcl-2. Irradiation increased the expression of pro-apoptotic proteins; p53, PUMA, Bax, cytochrome c, and cleaved caspase-9 and -3, whereas it decreased the expression of anti-apoptotic protein Bcl-2 (Figure 3D). Importantly, KGF-1 treatment significantly inhibited the irradiation-induced induction in expression of pro-apoptotic proteins and increased the expression of anti-apoptotic protein. In the presence of FGFR2 inhibitor to block endogenous KGF signaling, irradiation-induced apoptosis in hPECs was still observed, suggesting an anti-apoptotic effect of exogenous KGF-1. In sum, the protective effect of KGF-1 on irradiation-induced apoptosis in hPECs is associated with regulation of p53-mediated apoptosis pathway (Figure 3D).

To determine the signaling pathway, we next explored the expression of FGFR2 and its downstream signal transduction pathway. Given the role of PI3K-Akt pathway of growth factor signaling, we examined activation of PI3K, its downstream target, Akt, and an important p53 suppressor, murine double minute 2 (MDM2). Irradiation reduced the expression of PI3K-Akt-MDM2 axis (Figure 3E). Phosphorylations of PI3K, Akt, and MDM2 were enhanced in response with KGF-1 pretreatment. These results suggest that KGF-1 induces PI3K and Akt activation and subsequent phosphorylation of MDM2 inhibits irradiation-induced p53 activation. In the presence of PI3K inhibitor, the loss of Akt phosphorylation confirmed the efficacy of the inhibitor, and inhibition of MDM2 phosphorylation by PI3K inhibitor suggested that down-regulation of the anti-apoptotic pathway antagonized p53 and its downstream pro-apoptotic proteins (Figure 3F–3G). Furthermore, both inhibitors blocked the promotion of proliferative capacity and inhibition of apoptosis by KGF-1 (Figure 3H). These results provide support for the protection of KGF-1 by blocking apoptotic signaling pathway that is induced by known PI3K-Akt-MDM2 axis (Figure 3I).

### Morphological improvement, cytoprotection and anti-apoptosis following irradiation by KGF-1 treatment

**Figure 4:**
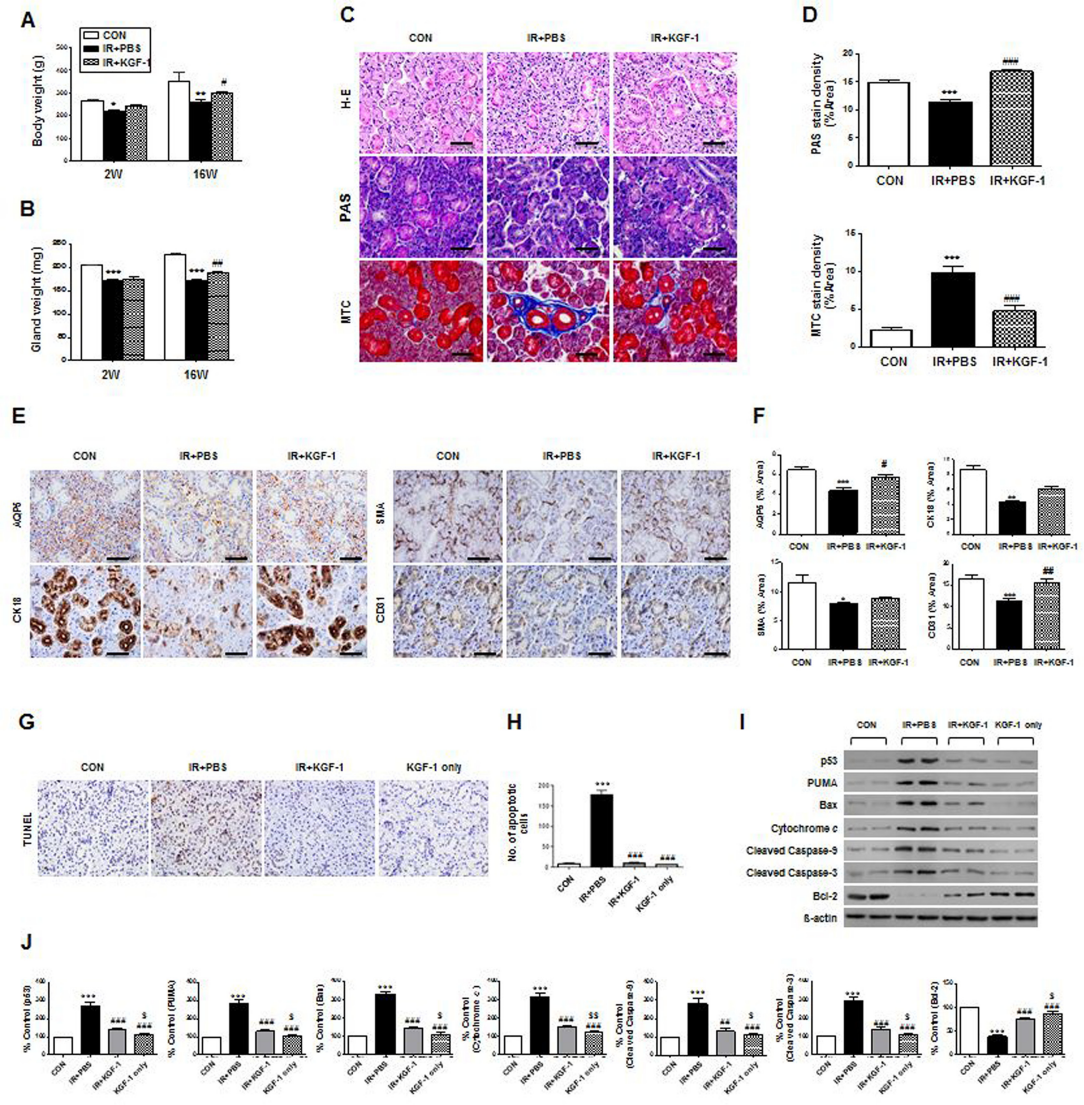
Macro- or micro-morphological evaluation, cytoprotective effect and anti-apoptosis of KGF-1 treatment (**A** and **B**) Body weight and dissected glandular weight were measured at two and 16 weeks after irradiation. Two-way ANOVA, Bonferroni's post hoc test. *, compared to CON in each group; ^#^, compared to IR+PBS in each group. *P <0.05, **P <0.01, ***P <0.001, ^#^*P* < 0.05, ^##^*P* < 0.01. (**C**) Representative histological pictures of H-E, PAS and MTC staining from three groups at 16 weeks post-irradiation are presented. Scale bars represent 50 μm. (**D**) Densities upon PAS (upper) staining were measured using a software program to calculate pixels of purple stained mucin-containing areas. MTC (lower) staining showed the IR+KGF-1 group exhibited less periductal and perivascular fibrosis than the IR group. One-way ANOVA, Tukey's post hoc test. *, compared to CON; #, compared to IR. ***P < 0.001, ^###^*P* < 0.001. (**E**) Representative images of salivary epithelial (AQP5), endothelial (CD31), myoepithelial (α-SMA), and ductal cells (CK18) in the SG in the three experimental groups. Scale bars represent 50 μm. (**F**) Each staining area was measured in pixels using a software program. Data are presented as the mean area (%) ± SEM. One-way ANOVA, Tukey's post hoc test. *, compared to CON; ^#^, compared to IR. *P < 0.05 **P < 0.01 ***P < 0.001, ^#^*P* < 0.05, ^##^*P* < 0.01. two weeks (*n* = 3 in all groups), 16 weeks CON group (*n* = 3), IR+PBS group (*n* = 3), IR+KGF-1 group (*n* = 2). Five random sections from each rat were evaluated by a blinded researcher. The number of slides examined from each experimental group ranged from 18 to 21. (**G**) Representative images of an *in vivo* TUNEL assay from three experimental groups at two weeks post-irradiation. Scale bars represent 50 μm. (**H**) The number of TUNEL-positive apoptotic cells was determined by a blinded researcher. Data are presented as the mean number of apoptotic cells per field ± SEM. One-way ANOVA, Tukey's post hoc test. *, compared to CON; ^#^, compared to IR. ***P < 0.001, ^###^*P* < 0.001. (*n* = 2 mice in all groups. Five random sections in each experimental group were examined.) (**I**) Representative images of apoptotic cascades as determined by Western blotting. (**J**) Comparison of p53, PUMA, BAX, cytochrome C, cleaved caspase-3, cleaved caspase-9 and Bcl-2 expression (results are presented as the mean ± SEM). One-way ANOVA, Tukey's post hoc multiple comparisons test. *, compared to CON; ^#^, compared to IR; ^$^, compared to IR+KGF-1. ***P < 0.001, ^##^*P* < 0.01, ^###^*P* < 0.001, ^$^*P* < 0.05, ^$$^*P* < 0.01. (*n* = 2 mice in all groups).

We determined that rat salivary epithelial cells *in vivo* demonstrated immunoreactivity to FGFR2 (data not shown). Next, we performed an *in vivo* experiment testing whether local administration of KGF-1 could ameliorate irradiation-induced salivary hypofunction. External appearance and dissected SGs in each group were observed at 16 weeks after irradiation. Neck irradiation resulted in loss of hair around the neck and decreased glandular size, but KGF-1-treated rats showed less hair loss and larger glandular size than the irradiation group. Body weight and dissected glandular weight were measured at two and 16 weeks after irradiation (Figure 4A and 4B). Body weight and glandular weight were significantly lower in the irradiation group than the control group at two and 16 weeks after irradiation. However, KGF-1 treatment led to significantly increased body and glandular weight compared to the irradiation group at 16 weeks after treatment.

Histological evaluations of the micro-morphological changes were performed and the densities of mucin were measured by PAS staining followed by examination by a blinded researcher using a software program to calculate the pixels of purple stained areas at 16 weeks after irradiation. The salivary acinoductal structure was destroyed and reduced after irradiation, but maintained in the KGF-1 group (Figure 4C). Morphometric analysis of PAS staining showed that mucin production decreased significantly after irradiation, but increased significantly in the KGF-1 group relative to the irradiation group (Figure 4C and 4D). MTC staining showed irradiation-induced fibrotic changes in peri-acinoductal area but less in the KGF-1 group (Figure 4C and 4D).

We next investigated the cellular effects of KGF-1 on salivary epithelial, endothelial, myoepithelial, and ductal cells (Figure 4E). Immunohistochemical staining revealed that expression of AQP5 (a marker of salivary epithelial cells), CK18 (ductal cells) CD31 (endothelial cells), and α-SMA (myoepithelial cells) was significantly lower in the irradiation group than the control group (Figure 4E and 4F). Treatment of the KGF-1 increased the staining density of these cells relative to the irradiation group, suggesting KGF-1 could preserve myoepithelial and endothelial cells as well as salivary epithelial cells against irradiation-induced cell depletion.

Next, we performed an *in vivo* experiment testing whether local delivery of KGF-1 could prevent irradiation-induced salivary hypofunction. Local irradiation at 15 Gy to the neck induced apoptosis in SGs, whereupon TUNEL positive apoptotic cells were markedly increased at 24 hours after irradiation (Figure 4G). Apoptotic cell death, however, was reduced by treatment with KGF-1 before and immediately after irradiation. Quantification of TUNEL positive cells revealed that a significant increase in apoptosis was observed after irradiation, but this decreased significantly following KGF-1 treatment relative to the irradiation (vehicle PBS treated) group (Figure 4H). KGF-1 alone without irradiation did not increase cell death. We examined the changes in apoptosis-associated proteins *in vivo* (Figure 4I and 4J). Similar to *in vitro* findings, irradiation increased the expression of pro-apoptotic proteins; p53, PUMA, Bax, cytochrome c, and cleaved caspase-9 and -3, whereas it decreased the expression of anti-apoptotic protein Bcl-2. KGF-1 treatment significantly inhibited the irradiation-induced induction in expression of pro-apoptotic proteins and increased the expression of anti-apoptotic protein. These findings suggest that the radioprotective effect of KGF-1 is associated with regulation of p53-mediated apoptosis pathway *in vivo*.

### Recovery of irradiation-induced salivary hypofunction

**Figure 5:**
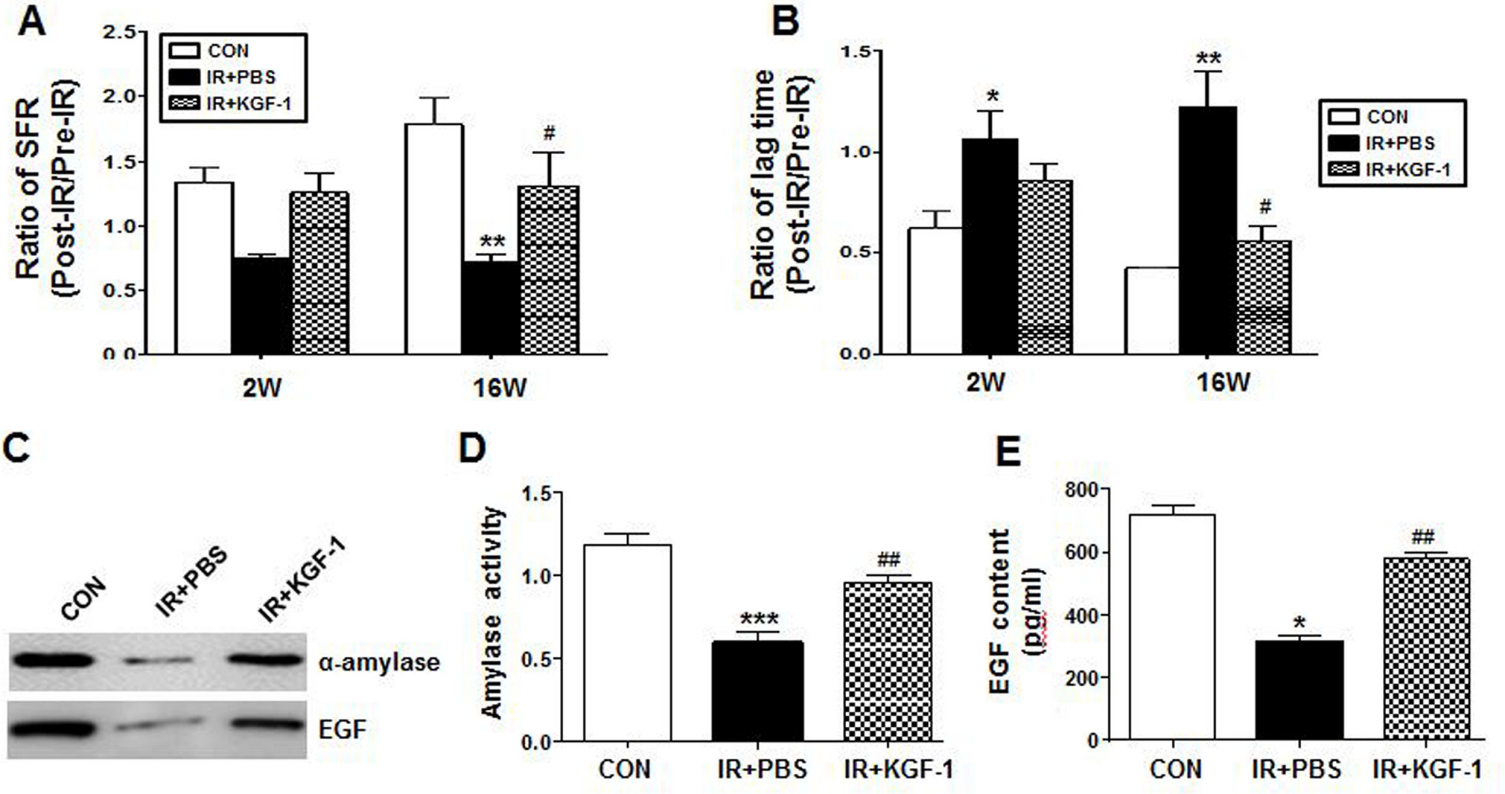
Effect of salivary function by KGF-1 treatment (**A**) Salivary flow rate (SFR) was calculated at pre-irradiation and 2 and 16 weeks post-irradiation. The changes in SFR after irradiation were expressed by the ratio of post-irradiation SFR to pre-irradiation SFR (Mean ± SEM). (**B**) Time to salivation (lag time, LT) was measured and the ratios of post-irradiation LT to pre-irradiation LT were presented. Data are presented as the mean ± SEM. (A, B and E) Two-way ANOVA, Bonferroni's post hoc test. *, compared to CON in each group; ^#^, compared to IR+PBS in each group. *P < 0.05, **P < 0.01, ^#^*P* < 0.05. (**C**) Western blotting of amylase and EGF in saliva at 16 weeks post-irradiation. (**D**) The salivary amylase activity was examined by the Assay Kit and fold changes in activity level are presented. (**E**) EGF contents were measured at each time point and the average contents are presented. Data are presented as the mean ± SEM. One-way ANOVA, Tukey's post hoc test. *, compared to CON; ^#^, compared to IR. *P < 0.05, ***P < 0.001, ^##^*P* < 0.01. 2 weeks (*n* = 3 in all groups), 16 weeks CON group (*n* = 3), IR+PBS group (*n* = 3), IR+KGF-1 group (*n* = 2).

The amount of salivation and lag time were measured pre-radiation and two and 16 weeks post-radiation. The changes in SFR and lag time after irradiation were calculated by the post- radiation to pre-radiation ratio. Irradiation significantly decreased the ratio of SFR at 16 weeks and increased the ratio of the lag time at two and 16 weeks after irradiation (Figure 5A and 5B). KGF-1 treatment significantly improved the ratio of SFR and the lag time at 16 weeks after irradiation. We performed western blotting of saliva collected at 16 weeks post-radiation to examine the salivary amylase and EGF protein expression level (Figure 5C). The expression levels of salivary amylase and EGF were higher after KGF-1 treatment relative to the irradiation group. The average activity of salivary amylase was significantly reduced in the irradiation group (Figure 5D), and it was significantly improved in the KGF-1 group. EGF contents in saliva at each time point were also confirmed by ELISA. The average concentration was significantly lower in the irradiation group than the control group (Figure 5E), and significantly increased in the KGF-1 group relative to the irradiation group at 16 weeks after treatment.

## DISCUSSION

In this study, we investigated the KGF-1 mechanism related to the anti-apoptotic effect *in vitro* and protective effects of local administration of KGF-1 on SG function following irradiation *in vivo*. With KGF-1 treatment, we verified the radioprotective mechanism of KGF-1 by protection against DNA damage and p53-mediated apoptosis through PI3K-Akt pathway after irradiation using human SG epithelial cell culture *in vitro*. Salivary function recovered within 16 weeks after irradiation and these findings are supported by an earlier study that delivered KGF-1 gene to submaxillary glands transductally in mice [5].

The exact mechanism of SG dysfunction induced by irradiation remains unclear. Irradiation causes DNA damage and generates reactive oxygen species, an important cause of apoptotic cell death in target tissues. Reactive oxygen species lead to apoptosis and to the up-regulation of inflammatory cytokines. The latter can produce further tissue damage, amplifying signaling cascades and the entire injury process [15]. One of the most consistent phenomena in SG response to irradiation is the loss of cellular components in our study. These findings imply that a continued apoptotic response contributes to these observations. Avila et al. suggested that the apoptotic response after irradiation is mediated by p53 and is directly associated with salivary dysfunction [16]. p53 induces apoptosis by transcriptional regulation of target genes, and involves mitochondrial cytochrome c release and caspase9 and -3 cascades [17]. A number of p53-regulated genes have been identified, and some of these represent potential downstream mediators of p53- dependent apoptosis including Bax and PUMA [18, 19]. The Bax protein accumulates in mitochondria in response to death signals [18]. PUMA localizes to the mitochondria and interacts with Bax. PUMA interaction with Bax rapidly induces apoptosis through cytochrome c release and activation of caspase-3 and -9 [19]. Irradiation increases expression of pro-apoptotic proteins and decreases expression of anti-apoptotic protein. KGF-1 treatment significantly decreases the irradiation-induced expression of pro-apoptotic proteins and increases the expression of anti-apoptotic protein.

PI3K/Akt pathway is important in cell survival and proliferation in cells [20, 21]. To addressing the role of KGF-1 to block the apoptosis in the model of hPECs, we focused on the PI3K/Akt signaling axis, a pathway with protective effects that inhibit cell apoptosis. We postulated that KGF-1 could induce PI3K, Akt, and MDM2 phosphorylation in hPECs. Phosphorylations of PI3K-Akt-MDM2 axis were enhanced in response with KGF-1 pretreatment. These results support our hypothesis that KGF-1 preserves hPECs viability by inhibiting apoptosis, probably by mediating the PI3K-Akt-MDM2 pathway. As a critical regulator of PI3K-mediated cell survival, activation of Akt signaling is sufficient to block cell death induced by apoptotic stimuli. The mechanisms underlying the cytoprotective effects of KGF-1 in epithelial cells are mediated via PI3K–Akt signaling [22, 23]. These findings are reproduced in our study that KGF-1 treatment reduced the extent of irradiation-induced DNA damage and apoptotic cell death in salivary epithelial cells via activation of the receptor tyrosine kinase (RTK), FGFR2IIIb and PI3K-Akt signaling transduction pathway. PI3K is a vital regulatory protein responsible for cell growth, proliferation, differentiation, motility, and survival and these functions are related to the ability to activate protein kinase B (Akt). Akt mediates anti-apoptotic action through phosphorylation of multiple downstream targets including MDM2, a p53 interacting protein that downregulates p53 transcriptional activity [24].

Salivary parenchymal cells have FGFR2 receptors that are required for KGF-1 signaling [6]. We first examined sections of SGs to determine whether they expressed FGFR2 and rat salivary parenchymal cells showed FGFR2, and it was distributed to ductal area. KGF-1 has the potential to protect many types of cells against irradiation [6, 10, 14, 25, 26]. Our results showed that the KGF-1 administered to SGs can prevent irradiation damage in acinar, ductal, endothelial and salivary stem/progenitor cells. Lombaert IM et al. demonstrated that expansion of salivary stem/progenitor cells was achieved by subcutaneous administration of KGF-1, resulting in the preservation of saliva secretion after irradiation in mice [5]. Transductal KGF-1 gene delivery to submaxillary glands successfully prevents salivary hypofunction by increasing proliferation of salivary parenchymal and endothelial cells [6]. Salivary microvascular endothelial cells might be early and sensitive targets of irradiation in both murine and porcine SGs [27, 28].

In this study, KGF-1 was delivered to SGs through local injection because of concern that systemic delivery of KGF-1 could produce a malignancy [29, 30]. Intraductal delivery of adenoviral vectors to rodent SGs has been shown to be safe [31–34]. However, intravenous delivery does not alter tumor response or patient survival [7]. Using murine xenograft models for head and neck and colorectal carcinoma, KGF-1 given subcutaneously had no effect on either tumor growth or antitumor chemotherapeutic activity [8]. To begin to address these concerns, this study examined the protective efficacy of KGF-1 when locally administered to irradiate SGs *in vivo*. We injected KGF-1 twice (1 hour before and immediately after irradiation) so that KGF-1 locally injected into SG could act as a mitigator of early radiation damage.

In summary, the present study shows that locally administrated KGF-1 into the irradiated SGs effectively protects SG subtype cells, preventing hypofunction of salivation *in vivo*. The PI3K-Akt- pathway mediates the anti-apoptotic effect of KGF-1 in salivary epithelial cells, and leads to activation of Mdm2 and blockade of the p53-mediated apoptotic pathway. Our results here clearly show that new strategies for development and design of novel effective therapeutic drugs based on the activation of KGF-1 signaling pathways are needed for treatment of patients with irradiated SG damage. Thus, KGF-1 may be beneficial to prevent irradiation-induced SG hypofunction in patients being treated for head and neck cancers.

## MATERIALS AND METHODS

### Human salivary gland epithelial cells culture

We obtained hPECs from a patient who underwent parotidectomy due to benign parotid tumor, as described in our previous study [35]. The specimen was collected with informed consent and institutional review board approval. A small portion of a non-tumor bearing gland was resected and washed thrice with cold 1×PBS containing 1% antibiotics. The tissue was finely chopped and enzymatically digested with 0.25% collagenase type B (2.5 mg/mL) and DNase I (1 mg/mL) with gentle shaking at 37°C for 30 minutes. The cell suspension was subsequently filtered through a 70 μm cell strainer, then centrifuged at 1500 rpm for five minutes, after which it was plated on a culture dish and cultured with Keratinocyte-Serum Free Medium (KSFM; Gibco, Grand Island, NY, USA) containing epidermal growth factor (EGF, 5 ng/ml), calcium chloride (CaCl_2_, 0.09 mM) and 1% antibiotics.

### *In vitro* irradiation and cell morphology

For irradiation experiments, the hPECs at passage 2–3 were seeded at 2 × 10^4^ cells/well, cultured at 37°C for three days on 6-well plates, and irradiated with 0, 10, and 20 Gy using a 4 MV X-ray from a linear accelerator (Mevatron MD, Siemens Medical Laboratories Inc., Germany). 100 ng/ml KGF-1 (R&D Systems, Minneapolis, MN, USA) was administered immediately after irradiation, cell morphology was analyzed under an inverted phase-contrast microscope (Olympus FV1000, Olympus, Tokyo, Japan), and images were obtained using a digital camera at one, two, and three days after irradiation.

### Cell proliferation, cytotoxicity, and viability

To investigate the cell proliferation of hPECs, an MTS (3-(4,5-dimethylthiazol-2-yl)-5-(3-carboxymethoxyphenyl)-2-(4-sulfophenyl)-2H-tetrazolium) uptake assay was used. HPECs were seeded at 1 × 10^4^ cells/well, cultured at 37°C for three days. The cultured cells were randomly divided into three groups: 1) Normal control group without irradiation and KGF1 treatment (CON), 2) irradiation without KGF-1-treatment (IR), 3) Irradiation with KGF-1-treatment (IR+KGF-1). The IR and IR+KGF-1 groups were irradiated with 15 or 20 Gy using 4 MV X-ray from a linear accelerator (Mevatron MD, Siemens Medical Laboratories Inc., Germany). KGF-1 (50 ng/ml or 100 ng/ml or 200 ng/ml, R&D Systems, Minneapolis, USA) was applied in KGF-1 added groups after irradiation. After addition of 20 μl MTS reagent and incubation at 37°C for four hours, proliferation of hPECs was investigated by reading the absorbance at 490 nm using a 96-well plate reader (Dynex Revelation, Dynex Ltd., Billingshurst, UK). At 1, 2, 3 days after irradiation, 100 μl of supernatant per well was harvested and transferred into a new 96-well, flat-bottom plate. LDH substrate (100 μl) was added to each well and incubated for 30 minutes at room temperature protected from light. The absorbance of the samples was measured at 490 nm with an ELISA reader. For assessment of proliferation and cytotoxicity following irradiation, at least three independent experiments were performed.

To assess the cell viability, a LIVE/DEAD assay kit (Invitrogen, CA, USA) was utilized according to the manufacturer's instructions. HPECs were seeded in 6-well plates at a density of 2 × 10^5^ cells per well and cultured for up to three days. Cells were washed with 1×PBS, detached using 0.5% Trypsin-EDTA, neutralized with cell culture media, and collected in 50 ml tubes. Next, cells were centrifuged at 1500 rpm for five minutes, the supernatant was discarded, and pellets were resuspended in trypan blue dye in media for one minute before counting using a hemocytometer.

### Evaluation of apoptosis, DNA damage

The cultured cells were randomly divided into five groups: 1) Normal control group without irradiation and KGF-1 treatment (CON), 2) Irradiation without KGF-1-treatment (IR), 3) Irradiation with KGF-1-treatment (IR+ KGF-1), 4) FGFR2 inhibitor added before irradiation and KGF-1 treatment after irradiation (IR+FGFR2 inhibitor), 5) PI3k inhibitor added before irradiation and KGF-1 treatment after irradiation (IR+PI3k inhibitor). For immunofluorescence staining, cells were washed and fixed in 4% paraformaldehyde for 20 minutes at room temperature, then permeabilized with 0.4% Triton X-100 in 1×PBS for 10 minutes at room temperature. After washing with 1×PBS, cells were treated with 1% BSA in 1×PBS for one hour before incubation with primary antibodies specific for anti-γ-H2AX (Abcam, Cambridge, UK) in 1% BSA overnight at 4°C. The cells were washed in PBS before incubation with a goat anti-rabbit IgG-Alexa-488-conjugated secondary antibody (Invitrogen, Camarillo, CA, USA) for six hours at room temperature in the dark. The nucleus was counterstained with 4′,6-diamidino-2-phenylindole, dihydrochloride (DAPI; Vector Labs), after which the cells were viewed using a confocal laser scanning microscope (Olympus FV1000, Olympus, Tokyo, Japan).

Effects of the FGFR2 inhibitor, LY2874455 (2 nΜ, Sellekchem, Boston, MA, USA), PI3k inhibitor, BKM120 (100 nΜ, Sellekchem, Boston, MA, USA) on the action of KGF-1 in irradiation-induced changes of hPECs were investigated. The hPECs at passage 2–3 were seeded at 2 × 10^4^ cells/well, cultured at 37°C for three days on 6-well plates, and irradiated with 0, 10, and 20 Gy using a 4 MV X-ray from a linear accelerator (Mevatron MD, Siemens Medical Laboratories Inc., Germany) before treatment with FGFR2 inhibitor (2 nM) and PI3k inhibitor (100 nM).

### Evaluation of salivary phenotypic gene expression

The levels of transcripts were determined by real-time PCR (RT-PCR) on the ABI PRISM sequence detection system using SYBR Green I as a double-stranded DNA-specific dye according to the manufacturer's instructions (Applied Biosystems, Foster City, CA, USA). The PCR reaction was carried out using 1 μM cDNA, 10 μM SYBR Green PCR master mix (Roche Diagnostics, Laval, Quebec, Canada), 10 pM sense and antisense primers specific for α-amylase (*AMY1A*), AQP5 (*AQP5*), E-cadherin (*CDH1*), ZO-1 (*TJP1*), CK7 (*KRT7*) and CK18 (*KRT18*) in a reaction mixture with a final volume of 20 μL (Table 1). The amount of real-time PCR products was normalized with the house-keeping gene, β-actin.

**Table 1:** Primers used for RT-PCR

Gene and symbol		Primer sequences (5′–3′)
α-Amylase (*AMY1A*)	F	AAT TGA TCT GGG TGG TGA GC
	R	CTT ATT TGG CGC CAT CGA TG
Aquaporin 5 (*AQP5*)	F	ACT GGG TTT TCT GGG TAG GG
	R	GTG GTC AGC TCC ATG GTC TT
E-Cadherin (*CDH1*)	F	CGC ATT GCC ACA TAC ACT CT
	R	TTG GCT GAG GAT GGT GTA AG
ZO-1 (*TJP1*)	F	TTT GGC CGA GGG ATA GAA GT
	R	TAT TGC CAT CTC TTG CTG CC
CK7 (*KRT7*)	F	CAG GAT GTG GTG GAG GAC
	R	AAC TTG GCA CGC TGG TTC T
CK18 (*KRT18*)	F	GGA GGC TGG AGA GCA AAA TC
	R	AGT CAT CAG CAG CAA GAC GG

### Western blotting

For western blot analysis, samples were isolated from the lysate (30 μg), mixed in reducing buffer, boiled, resolved on sodium dodecyl sulfate polyacrylamide gel electrophoresis, and transferred to a polyvinylidene difluoride membrane by blotting. The blot was incubated overnight at 4°C in a blocking solution with primary antibodies to the following antigens: α-amylase, AQP5, ZO-1, CK7, CK18, FGF7, FGFR2, p53 upregulated modulator of apoptosis (PUMA) and β-actin (Santa Cruz Biotechnology, Santa Cruz, CA, USA); E-cadherin (BD Pharmingen, San Diego, CA, USA); p53, Bax (Abcam, England); Bcl-2, Cytochrome C, Cleaved caspase-9 and Cleaved caspase-3, protein kinase B (PI3K), p-PI3K, Akt, p-Akt and murine double minute 2 (MDM2), p-MDM2 (Cell signaling, Danvers, MA, USA). After washing the blots with 0.1% Tween 20 in 1×PBS, they were incubated with horseradish peroxidase-conjugated secondary antibodies corresponding to each primary antibody, after which they were subjected to enhanced chemiluminescence detection (GE Healthcare Life Science, USA).

### Animal experiments

This study was approved by the Animal Ethics Committee of Inha University Hospital. Animals were cared for in accordance with established institutional guidelines. Female SD rat (*n* = 24) weighing 280–350 g were used for the experiments. (Orient Bio, Gyeonggi-Do, Korea) The animals were kept under clean, conventional conditions. They were given access to standard laboratory food and sterilized water ad libitum. Rats were anesthetized with xylazine (10 mg/kg, i.p) and ketamine (100 mg/kg i.p) and fixed in a plane board. To cause irradiation-induced SG hypofunction, irradiation was carried out with 4 MV X-ray emitted from a linear accelerator (Mevatron MD, Siemens Medical Laboratories Inc., Germany) with a single dose of 15 Gy at a focus-to-skin distance of 100 cm. Rats were irradiated in the head and neck field with the body shielded from irradiation.

KGF-1 was dissolved in PBS containing 0.1% BSA (100 μg KGF-1/ 1 ml PBS). A 5-mm incision to the left neck of the rats was made to expose ipsilateral submaxillary gland. KGF-1 (20 μl) or PBS solution (20 μl) was injected twice; 1 hour before irradiation and immediately after irradiation directly to the submaxillary glands using a syringe with a 21-gauge needle. After injections, the neck wound was sutured and sterilized. Vehicle (PBS)-treated rats were served as an irradiation control group. KGF-1-injected without irradiation group was included to determine its mitogenic effect.

### TUNEL assay

Apoptotic cells dissected glands at 24 hours after treatment were visualized using the Apoptag Plus Fluorescein *In Situ* Apoptosis Detection kit (Millipore, Bedford, MA, USA), and two blind examiners independently counted the absolute number of apoptotic cells in three random fields per tissue section under 400 × magnification. At least three random tissue sections per gland were chosen for each slide.

For *in vitro* TdT-mediated dUTP nick-end labeling (TUNEL) staining, cells were washed and fixed in 4% phosphate-buffered paraformaldehyde for 25 minutes at 20°C. After washing thrice with PBS, cells were stained using the *In Situ* Cell Death Detection Kit (Roche Diagnostics, Laval, Quebec, Canada) according to the manufacturer's protocol. The nucleus was counterstained with DAPI, and the cells were visualized under the Axiovert 200 fluorescence microscope with 10 × 10 and 20 × 10 NA objectives equipped with AxioCam HRC digital camera.

### Morphological, histological, and immunohistochemical evaluation

Body weights were measured at two and 16 weeks after irradiation followed by saliva collection and humane euthanasia. The submaxillary glands of the mice were harvested and the surrounding fat and connective tissues were removed. The glandular weights were measured and submaxillary glands were immediately placed in 4% paraformaldehyde at room temperature.

To evaluate the functionality of SG components, tissues were processed, embedded in paraffin, sectioned at 4 μm, and stained with hematoxylin-eosin (H&E), Masson's Trichrome (MT) and Periodic Acid Schiff (PAS). The sections were washed in phosphate buffered saline (PBS) and pre-incubated for one hour in a blocking solution containing 5% normal goat serum in PBS. After a brief PBS wash, primary antibodies for aquaporin (AQP)-5 (Alomone labs, Jerusalem, Israel), cytokeratin (CK)-18 (Santa Cruz Biotechnology, Texas, USA), α-SMA and CD31 (Abcam, Cambridge, UK) diluted in blocking solution were applied. Sections were incubated overnight at 4°C, in secondary antisera (Invitrogen, Camarillo, CA, USA) for 90 minutes at room temperature, and treated with avidin–biotin–peroxidase solution. The peroxidase label was visualized using diaminobenzidine as the chromogen (Vector Laboratories, Burlingame, CA, USA). Sections were washed, allowed to air dry, cover slipped, and processed for observation under a confocal microscope (IX81, Olympus, Center Valley, PA, USA). Three sections were made for each gland, three random fields per section of gland were checked by two blind examiners, and positive cell numbers or stained areas were measured using MetaMorph software (Molecular Devices Corporation, Sunnyvale, CA, USA).

### Salivary function evaluation

Salivary secretory function was determined by measuring salivary flow rate (SFR) and lag time of salivation at two and 16 weeks after irradiation. Saliva was collected using a micropipette for 10 minutes after stimulation by intraperitoneal injection of pilocarpine (2 mg/kg) when rat salivated in the floor of mouth. Collected saliva was placed in pre-weighed 1.5 ml micro-centrifuge tubes, and SFR (μl/ml) was calculated as the total saliva weight (mg) divided by the collection time (minutes) (saliva has a specific gravity of 1 mg/ml). The lag time of salivation was determined as the time from stimulation to the beginning of saliva secretion. To reduce the effects of diurnal variation, saliva was collected consistently at 2:00 p.m.

### Amylase and EGF contents in saliva

Saliva samples obtained at 16 weeks after irradiation were centrifuged at 6000 rpm for 15 minutes. Total supernatant protein concentrations were determined by bicinchoninic acid (BCA) assay (Pierce, Rockford, IL, USA), after which 1 μg of total saliva protein was separated on SDS-PAGE and transferred to an Immobilon nitrocellulose membrane (Millipore, Billerica, MA, USA). Next, the membrane was blocked with 5% skim milk/TBS for one hour at room temperature, then rinsed with wash buffer and incubated with mouse anti-amylase antibody (Santa Cruz Biotechnology, Santa Cruz, CA, USA) at a 1:5000 dilution. Following overnight incubation with shaking at 4°C, the membranes were rinsed with wash buffer thrice for five minutes each. This was followed by incubation with a 1:5000 dilution of anti-mouse IgG horseradish peroxidase conjugate (Santa Cruz Biotechnology, Santa Cruz, CA, USA) for one hour at 37°C. Finally, samples were rinsed with wash buffer as above, after which detection was conducted using enhanced chemiluminescence (ECL) western detection reagents (Elpisbio, Daejeon, Korea). The bands of interest were detected using a luminescent image analyzer (4000 r, Kodak, Rochester, NY, USA), and the results were quantified using a software program (Kodak).

In addition, amylase activity of secreted saliva was determined using a salivary α-amylase assay kit (Salimetrics LLC, State College, PA, USA) with 2-chloro-p-nitrophenol linked with maltotriose as the chromogenic substrate according to the manufacturer's instructions. The amount of α -amylase activity present in the sample is directly proportional to the increase in absorbance at 405 nm observed using a standard laboratory plate reader.

Saliva EGF was measured by enzyme-linked immunosorbent assay (ELISA) using a commercial kit (Quantikine; R&D Systems, Minneapolis, MN, USA) according to the manufacturer's instructions. The kit uses a sandwich ELISA that recognizes mouse EGF and has no detectable cross reactivity with other cytokines. Each sample from each group collected at different time points was assayed in duplicate and the plate was read at 450 nm. The EGF concentration of the samples was determined by reading the optical density of the sample against the values of the standard curve.

### Statistical analysis

Statistical analysis was conducted using the Graph Pad Prism 5 package (GraphPad Software Inc., La Jolla, CA, USA). The Mann-Whitney test was used to assess differences between groups. One way ANOVA followed by Tukey's post hoc test and two-way ANOVA followed by the Bonferroni post hoc test were used to compare values among groups. Linear regression was applied to evaluate the association between parameters. Null hypotheses of no difference were rejected if *p*-values were less than .05.
